# Peridynamic Modeling of Ruptures in Biomembranes

**DOI:** 10.1371/journal.pone.0165947

**Published:** 2016-11-09

**Authors:** Michael Taylor, Irep Gözen, Samir Patel, Aldo Jesorka, Katia Bertoldi

**Affiliations:** 1 Department of Mechanical Engineering, Santa Clara University, Santa Clara, California, United States of America; 2 School of Engineering and Applied Sciences, Harvard University, Cambridge, Massachusetts, United States of America; 3 Department of Chemistry and Chemical Engineering, Chalmers University of Technology, Göteborg, Sweden; University of Cambridge, UNITED KINGDOM

## Abstract

We simulate the formation of spontaneous ruptures in supported phospholipid double bilayer membranes, using peridynamic modeling. Experiments performed on spreading double bilayers typically show two distinct kinds of ruptures, floral and fractal, which form spontaneously in the distal (upper) bilayer at late stages of double bilayer formation on high energy substrates. It is, however, currently unresolved which factors govern the occurrence of either rupture type. Variations in the distance between the two bilayers, and the occurrence of interconnections (“pinning sites”) are suspected of contributing to the process. Our new simulations indicate that the pinned regions which form, presumably due to Ca^2+^ ions serving as bridging agent between the distal and the proximal bilayer, act as nucleation sites for the ruptures. Moreover, assuming that the pinning sites cause a non-zero shear modulus, our simulations also show that they change the rupture mode from floral to fractal. At zero shear modulus the pores appear to be circular, subsequently evolving into floral pores. With increasing shear modulus the pore edges start to branch, favoring fractal morphologies. We conclude that the pinning sites may indirectly determine the rupture morphology by contributing to shear stress in the distal membrane.

## Introduction

Mechanical stress typically causes biomembranes to form pores or ruptures. It was recently shown that a double phospholipid bilayer membrane, forming spontaneously from a lipid source on a solid support, can form both floral and fractal ruptures[1]. In double bilayer membranes, the two individual stacked bilayers are in close proximity, separated by a nanoscopically thin water layer. The original publication suggested that the differences between the two established rupture modes in double bilayers can be related to the thickness of the water layer in different regions of the double bilayer, as well as to the occurrence of interconnecting (“pinning”) sites, formed by complexation of divalent calcium ions by lipid headgroups between the two stacked membranes. However, the very small fluid volume entrapped between the bilayers, which is on the order of femtoliters, poses limits on experimental investigation of rupturing. The presence of pinning could only be confirmed indirectly by studying the effects of its reversal on the fractures[2]. Better understanding of the two rupture mechanisms, which is expected to yield insights into the conditions and structural requirements for fracture formation in biological membranes, clearly requires innovative approaches, including both models and experimental designs.

A typical lipid bilayer membrane is only 4–5 nm thick, but can extend many hundreds of micrometers laterally. With respect to fundamental material properties of double bilayer lipid films, they can be considered to be two stacked elastic sheets, with a large bending modulus (10–20 *k*_*b*_.*T*)[3], but comparatively small stretching capabilities (maximum 5% of its surface area)[3]. Phospholipid biomembranes are commonly described as two-dimensional fluid, thus their shear modulus is assumed to be G = 0. However, under certain conditions, e.g., lipid rafts in gel or solid phase, where the membrane is no longer fully fluid, lipid membranes can locally resist shear deformations and adopt non-zero shear moduli[4].

In an attempt to model, and consequently understand the rupture dynamics of such membranes, we have applied a peridynamics approach. Peridynamic theory is a reformulation of classical continuum elasticity theory that is particularly well-suited to the numerical modeling of rupture[5–7]. This approach has been used to model complex fracture phenomena in traditional engineering materials such as concrete[8–10], glass[11–13], composites[13–17], and metal[18–20]. Its application to the study of biological membranes at the micro-scale is novel and provides an exciting new avenue of inquiry.

The defining attribute of the peridynamic theory is that the governing equation of motion is integral rather than differential, as in the classical continuum theory. Particles are connected to a local neighborhood of adjacent particles via bonds. In other words, peridynamics is a non-local theory and can be thought of as an upscaling of molecular dynamics[21–22]. Material damage and fracture is handled constitutively at the bond level, eliminating the need for special crack initiation and growth algorithms found in standard numerical approaches (e.g., the finite element method[13]). Cracks in peridynamics form and move spontaneously along arbitrary paths as a natural outgrowth of the model. As a body deforms as a result of applied loading, individual bonds stretch. If a bond stretches beyond a critical value, it is broken and no longer carries any load. As a result, loads are redistributed among remaining bonds. In this way, cracks nucleate and grow autonomously as bonds break throughout the body (see S1 File for a more detailed introduction to peridynamic theory).

## Methods

### Experiments

**Fig 1:**
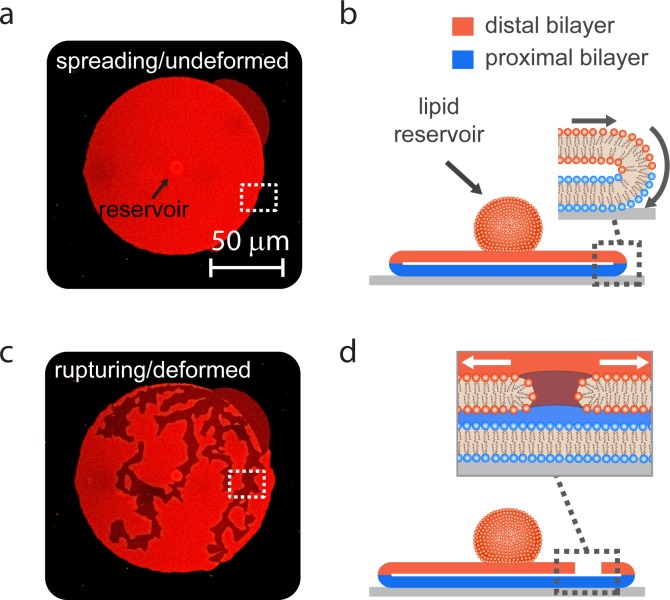
The experiment. (a) Confocal laser scanning micrograph of a spreading double lipid bilayer membrane(DLBM), top view. (b) Schematic drawing of the DLBM in (a), side view. DLBM consists of a distal (upper, red color) bilayer and the proximal (lower, blue color) bilayer. The spreading edge of the double bilayer performs a 'tank-tread' motion. (c) Micrograph of a ruptured membrane. (d) Schematic drawing showing a rupture in the distal membrane. Upon rupturing, the lipid material migrates towards the edges onto the substrate.

Lipid film precursors, buffered solutions, and high energy surfaces were prepared as described earlier[1]. As shown in Fig 1, Multilamellar (or onion shell) lipid vesicles were manually deposited on a silicon oxide surface, where self-spreading occurred spontaneously, forming a circular double bilayer patch (Fig 1A) with a fixed proximal (lower) and mobile distal (upper) bilayer (Fig 1B). In this tension-driven wetting process, the lipid reservoir is nearly fully consumed, at which point the rupturing is observed (Fig 1C and 1D). Spreading and rupturing of membranes were observed with a laser scanning confocal microscope (Leica TCS SP2 RS) using a 40x NA 1.25 oil objective. The membrane stain TR-DHPE was excited with a He/Ne laser at 594 nm. The emission was collected between 600–700 nm with a photomultiplier tube. The time series of spreading membranes were recorded with 0.62 Hz sampling frequency. Representative rupture processes recorded during our experiments are shown in S1–S3 Movies.

### Numerical Simulations

Although the phospholipid membranes we consider in this work have a double bilayer structure, the ruptures only appear in the mobile distal (upper) layer. Thus, in all our simulations we only model the expanding distal lipid layer as a two-dimensional membrane. We capture its interconnection with the proximal (lower) layer (i.e. the pinning sites) by fixing some material points in their initial positions for the duration of a particular simulation. Moreover, we also neglect the effect of the fluid surrounding the membrane. In fact, even in case of fully fluid-surrounded membranes, for example in vesicles, dissipation due to the flow of the membrane will dominate the pore dynamics compared to the dissipation caused by the water flowing through an opening pore, since the membrane is more viscous compared to water[23].

**Fig 2:**
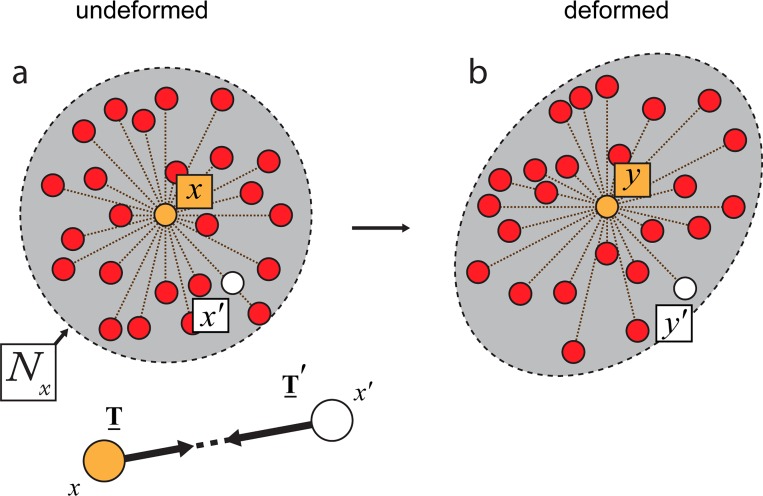
The peridynamic model. (a) A small, randomly selected region in the distal membrane is represented as a collection of particles (small circles). Each particle represents a collection of lipid molecules, and is located at the center of a circular neighborhood (N_x_). The motion of an arbitrary particle x (in yellow) at the center of N_x_ is influenced by the motion of every particle in N_x_ via bonds. If no forces apply to the membrane, the particles in N_x_ are considered to be in an undeformed state. The close-up shows vector ξ, representing the distance between bonded particles x and x’, where T is the force vector state that existed prior to the bond being broken. (b) As tension increases, the particles move apart from each other and the corresponding bonds stretch. At some critical value of stretch, the distance between the center particle (yellow dot) and some number of neighboring particles becomes too large, leading to broken bonds and disconnected particles (x', white dots). This corresponds to the rupture (pore) formation among membrane lipids.

We use a specially designed peridynamic code with a numerical implementation based on the state-based Linear Peridynamic Solid (LPS) model and algorithms used in the Sandia National Lab code LAMMPS [24], with key differences described below. Because of the thinness of the distal layer relative to its planar dimensions, practically being a molecular lipid film, we model it as a two-dimensional continuum of particles (i.e., material points) (Fig 2A) where each particle represents a number of lipid entities, not an individual molecule. More specifically, we use a two-dimensional approximation of the equation of motion (S1 File, Eq SI 1) [25] [20],

$$\int_{Nx}\left\{\underline{T}\left\langle x^{\prime }-x\right\rangle -\underline{T^{\prime }}\left\langle x-x^{\prime }\right\rangle \right\}dA_{x^{\prime }}=\rho \ddot{u}$$ (1)

where *ϵ* is the thickness of the membrane, *ρ* is the mass density, and $\underline{T}$ and $\underline{T\prime }$ are force vector states. A given particle, initially located at ***x***, is bonded to a certain number of its nearest neighbors, ***x***′, within a specified circular neighborhood *N*_*x*_ of finite radius *δ* (Fig 2A). The force vector state $\underline{T}$ (determined constitutively) acts on a bond with initial relative distance vector ⟨***x***′ – ***x***⟩ to yield the force density vector that ***x***′ exerts on ***x***. The corresponding force density vector that ***x*** exerts on ***x***′ is given by ${\underline{T}}^{\prime }\langle x-x^{\prime }\rangle$. The contributions of the force densities of all ***x***′ bonded to ***x*** are integrated over the area of *N*_*x*_, yielding the total force acting on the particle ***x***. The particle’s displacement is represented by the vector ***u***.

To discretize Eq 1 in space, we consider the membrane to be a finite collection of material points. We replace the integral in Eq 1 with a finite sum [26]:

$$\epsilon \;\sum_{j=1}^{n}\left\{\underline{T}\langle x_{j}-x_{i}\rangle -{\underline{T}}^{\prime }\langle x_{i}-x_{j}\rangle \right\}\;A_{j}=\rho {\ddot{u}}_{i}.$$ (2)

Thus, a material point *x*_*i*_ is bonded to all those *x*_*j*_ within its neighborhood *N*_*x*_. The quantity *A*_*j*_ is the area represented by material point *x*_*j*_ and is taken to be *h*^2^, where *h* is the initial particle spacing. In order to allow for rupture initialization, we specify a critical bond breaking stretch. An individual bond stretched beyond the critical value is broken (i.e., no longer able to carry load)(Fig 2B). Here, the critical bond breaking stretch is taken to be 15% throughout this study as it yields ruptures with similar qualities to the experiments.

In the standard peridynamic constitutive formulation used in this work, unphysical deformation modes allowing matter interpenetration are permitted[27]. This is different from constitutive theory in classical elasticity where interpenetration costs an infinite amount of strain energy. To address this issue, we specify short-range repulsive forces[22]:

$$f=min\left\{0,\frac{c\kappa }{\pi \delta^{5}}(\|y_{j}-y_{i}\|-d_{pi})\right\}\frac{y_{j}-y_{i}}{\left\|y_{j}-y_{i}\right\|},$$ (3)

where *d*_*pi*_ is the short-range interaction distance, *δ* is the neighborhood radius, and *c* is a constant to scale the force. In our simulations, *c* = 2.7*e* − 8 yielded good results. To prevent the unphysical particle overlap, we take[22]:

$$d_{pi}=min\{0.9\|x_{j}-x_{i}\|,1.35h\}.$$ (4)

A standard approach to temporal discretization of peridynamic models is to use explicit integration, e.g., velocity Verlet[20] or central differences[28]. These methods work very well for simulations of short time frames—those for which the small time steps required for stability in explicit methods are tolerable. Examples include high-velocity impact[22, 28–29] and blast loading[30–31]. In contrast, the problem of lipid bilayer spreading and rupturing unfolds over the course of tens or hundreds of seconds. Explicit integration would lead to prohibitively long simulation times. There are a couple of possible alternatives: quasi-static simulation[28, 32] or implicit integration. In this work we choose the latter and use the trapezoidal rule embedded in the adaptive time-step framework[33]. This method uses fixed point iteration, rather than Newton's method to solve for the positions at each step. In a nonlinear fracture problem, such as the one considered here, the stable time-step size is itself a function of time. The adaptive time-step assures convergence of the fixed-point iteration. In addition to implicit integration, we employ two other means of reducing the simulation time: mass scaling (in our simulations we take the membrane density to be 1000 *kg*/*m*^3^ scaled up by a factor of 10^8^) and GPU parallelization[19, 34]. The effect of mass scaling is described in S1 File.

In all of our simulations, we model the membrane as circular, with a diameter of 200*μm* in its initial state, and a thickness of 5*nm*. The material point spacing *h* is 1*μm* and the neighborhood radius *δ* is 3*μm*. This ratio of *δ* to *h* results in a good balance between numerical fidelity and computational efficiency [22]. Decreasing the material point spacing does not yield superior results in comparison with experimental data. The maximum number of fixed-point iterations is taken to be 8 and the tolerance is 10^−6^. Typical stable time-steps (for the chosen mass scaling) are approximately 10^−4^*s* prior to rupture and 10^−6^*s* during rupture. Decreasing the tolerance leads to a reduction in the time-step size but not to appreciable change in the simulation results. For computational efficiency, only those particles within a radius of 6 times the material point spacing can contribute short-range forces to a given particle. The tank-tread motion at the boundary (Fig 1B) motivated our choice to model the expansion of the membrane by applying a velocity (directed radially outward) to material points within a layer 10*μm* from the outer edge. Boundary points are more weakly connected than points in the bulk due to the presence of fewer bonds[28]. Applying loading to a boundary layer is a way of mitigating unphysical boundary fracture at the beginning of the simulation. Experimental measurement suggests that a constant expansion rate of 200*μm*/*s* is a reasonable choice for the boundary, which is what we use for all of the simulations in this study.

To model material behavior, we use a bulk modulus of 10 *MPa* [35] and consider shear moduli within the range of 0 *MPa to* 10 *MPa*, which corresponds to a Poisson’s ratio ranging from 0.13 to 0.5. This range of Poisson’s ratio is typical for the transition between incompressible (fluidic) and solid- or gel-like materials, as shown in a recent computational study[36]. It is important to note that previous work connecting peridynamics to classical linear elastic fracture mechanics yields a critical stretch that is a function of the elastic moduli, neighborhood radius, and the critical strain energy release rate [7]. We are not certain this model is appropriate for lipid bilayers. If it is, then our choice of fixing the critical stretch, bulk modulus, and neighborhood radius while varying the shear modulus effectively varies the critical strain energy release rate, too. Since we do not know an appropriate value for the release rate, nor how it depends on the fluidity of the lipid, we have decided to allow it to change in accordance with a fixed critical stretch.

## Results

**Fig 3:**
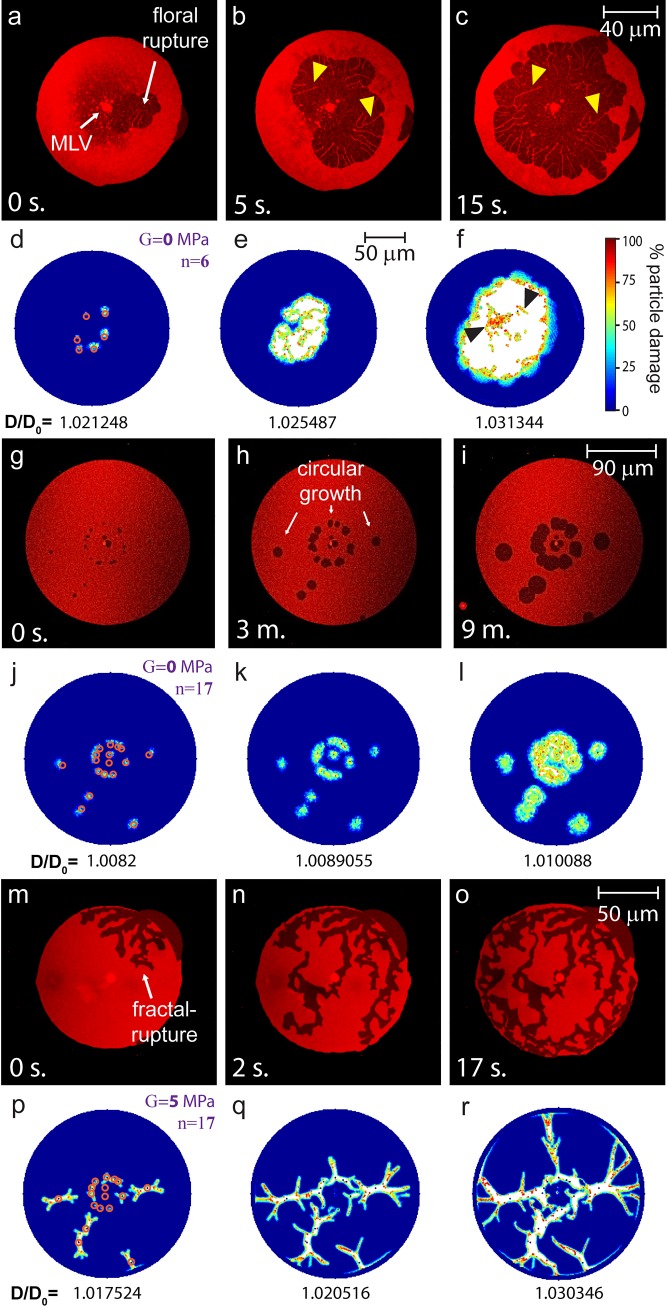
Floral and fractal biomembrane ruptures and corresponding peridynamic model simulations. (a-c) Confocal micrographs of a floral rupture occurring in the distal bilayer of a DLBM. Yellow arrow heads indicate threads of lipids between two layers, which are the pinned regions. (S1 Movie) (d-f) Peridynamic simulations showing floral ruptures (G = 0 MPa). The ruptures nucleate at the pre-determined locations of pinned (fixed) particles, and then merge into one large floral pore. The pinning points (n = 6) are marked with red circles in (d). Black arrow heads show threads of points remain between two layers which correspond to the pinned regions, similar to (c). (g-i) Confocal micrographs showing small circular pores opening and progressing in the distal bilayer. (S2 Movie) (j-l) Peridynamic simulations showing circular pores opening over time. Shear modulus G is 0 MPa as in (a-f), with the number of pinning points increased (n = 17). (m-o) Confocal micrographs of fractal ruptures occurring in the distal bilayer. (S3 Movie) (p-r) Peridynamic simulations showing fractal ruptures (G = 5 MPa). The number and location of pinning points are the same as in (g-l). The color bar in f applies to all simulations in Fig 3 and shows the amount of material point damage (%) where 100% damage corresponds to a complete breaking of all bonds associated with the material point. The scale bar in (e) applies to all simulations in Fig 3. The ratio of the diameter of the expanded membrane to the initial diameter (D/D_0_) is shown below each snapshot of the simulations.

In the experiments, ruptures reproducibly display two distinct morphologies: floral (Fig 3A–3F) and fractal ruptures (fractures, Fig 3M–3R). To determine which factors govern the occurrence of either rupture type we performed a number of numerical simulations, where we systematically changed both the number *n* of pinned particles within the expanding body and the shear modulus *G* of the membrane.

We first focus on the effect of the pinning sites, which in the experiments are induced by Ca^2+^ ions bridging the stacked bilayers locally. These pinning locations were earlier suspected to influence pore formation, and water content in different regions between the bilayers was linked to rupture morphology. In the experiments, direct evidence for the occurrence of pinning sites is derived from clearly visible thin membrane threads, left behind on the proximal bilayer by the rupturing distal membrane (as pointed by the yellow arrows in Fig 3B and 3C).

To better understand the effect of the pinning sites, we investigate numerically rupture in expanding membranes with G = 0 and different numbers of pinned points, n. In Fig 3D–3F and 3J–3L we present results for n = 6 and 17, respectively, with the pinning sites randomly distributed and marked with red circles. The snapshots indicate that the origin of the fracture events coincides precisely with the pre-defined pinning positions. With an increasing number of pinning sites, the number of nucleating pores are found to increase consistently. Moreover, if the pinning sites are concentrated within a small region of the membrane, they merge rapidly to form and progress as a floral pore (Fig 3D–3F). Differently, if the pinning sites are scattered throughout the distal membrane the pores individually grow over time, until the edges of the two pores meet and merge (Fig 3G–3L).

While the results of Fig 3D–3F and 3J–3L show that the pinning sites act as nucleation sites for the ruptures, they do not reveal a relationship between the fracture morphology and the location or number of pinning sites, as in both cases the rupture displays floral morphology. However, assuming that the pinning sites cause a non-zero shear modulus G, we observe a shift in pore morphology towards more elongated, channel-like morphologies, which resemble the fractal pores obtained in the experiments (see Fig 3P–3R for G = 5 MPa).

**Fig 4:**
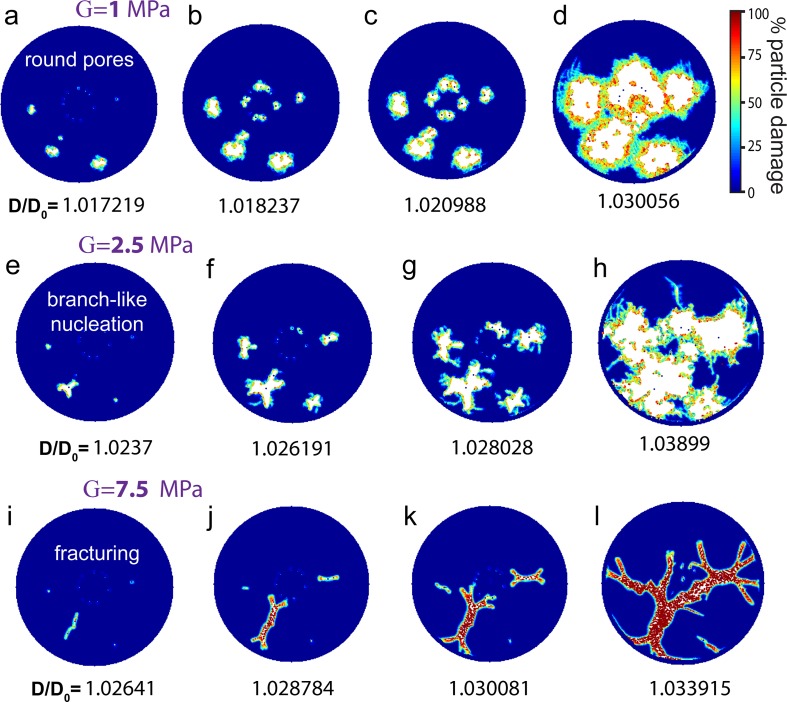
Transition in rupture morphology with increasing shear moduli. (a-l) The peridynamic simulations of the lipid membrane which is shown in Fig 3 (j-l), but with gradually increased shear modulus. (a-d) The ruptures become rugged where G = 1 MPa. (e-h) The straight edges of the ruptures become more pronounced where G = 2.5 MPa and branches start to appear. (i-l) The ruptures appear as elongated finely branched structures where G = 7.5 MPa. These structures typically evolve into fractals(l). The color bar in (d) applies to all simulations shown in Fig 4, and is identical to the one in Fig 3. The number of pinning points in all simulations in this figure is 16, and the positions of the pinning sites are identical to the ones in Fig 3J and p (S2 Movie). The ratio of the diameter of the expanded membrane to the initial diameter (D/D_0_) is shown below each snapshot of the simulations.

In order to study the effect of an increasing shear modulus on the rupture morphology, we altered G gradually, while keeping all other model parameters (including the quantity and the exact position of the pinning points) unchanged. The snapshots in Fig 4A–4D show that when we increased the shear modulus from G = 0 MPa to G = 1 MPa, the circular pore perimeters start to appear rugged. When G is increased further from 1 MPa to 2.5 MPa, the pore edges start to branch (Fig 4E–4H). This is a state where the ruptures do not look fractal, but their edges adopt features that are too irregular to still be considered as floral patterns. At G = 7.5 MPa the membrane pores appear as elongated, fine, branched structures (Fig 4I–4L). A very similar development can be observed for the fractal pores in Fig 3P–3R, where G = 5 MPa. In summary, Fig 4 shows that with increasing shear modulus the membrane pores transform from floral to fractals.

**Fig 5:**
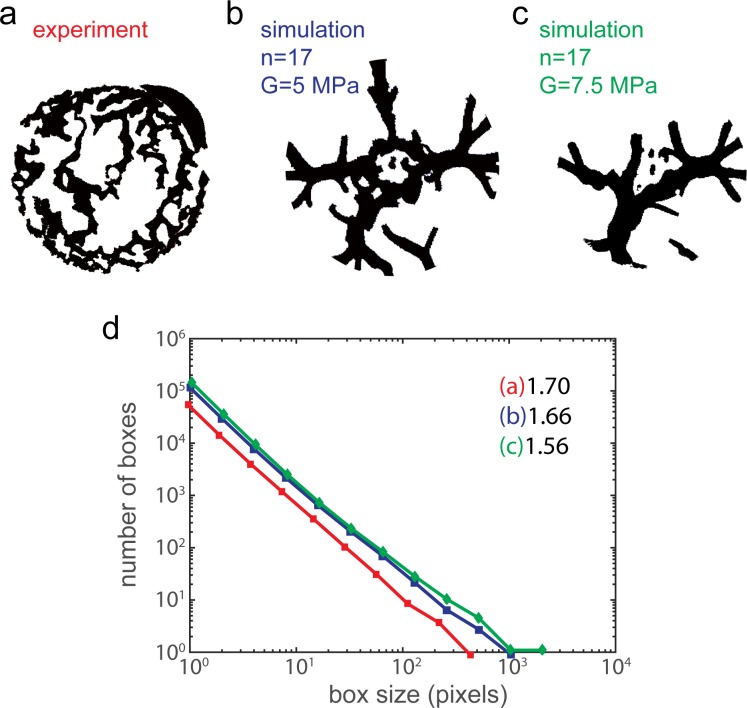
Fractal dimension analysis of ruptures in actual membranes and simulations. (a-c) Binary images showing the contour of the fractal ruptures in Fig 3O and 3R (G = 5) and 4l (G = 7.5). (c) Plots showing the fractal dimension (D) of rupture patterns in (a),(b) and (c). The slope of the red line shows the fractal dimension of the pattern in panel a, D = 1.63, the pattern in panel (b), D = 1.70, and the pattern in c, D = 1.56. The circular rim forming around the expanding membrane in the simulations has been removed manually with image processing software. All fractal dimensions have been calculated by using the reticular cell counting (box counting) method. The plots show the relation between the number of occupied boxes (y-axes) and the box size. The fractals in biological membranes (not shown) feature D values around 1.7[1]). The analysis from the simulations show that both slope and D are similar to the experimental values.

To be able to characterize the complex fractures in actual and simulated membranes, we calculated their fractal dimensions (D) using the box counting or grid method[37–38]. Briefly, an image is broken into smaller and smaller, squared shaped pieces, referred to as 'boxes'. A set of boxes is used to cover the image where each set has a different box size. For an image which contains a fractal pattern, this will result in 2 types of boxes in each set: those which contain a piece of the fractal image, and empty ones capturing only the empty background. The number of boxes that includes a piece of the fractal image is recorded as a function of box size. This relation, in logarithmic scale, gives a linear plot, whose slope is the fractal dimension D. In Fig 5 we compare D as calculated for the fractal patterns observed in the experiments and predicted by the simulations. The results indicate that the fractal ruptures forming in simulated membrane sheets display fractal dimensions comparable to those which are obtained for fractures observed in the experiments. In fact, for the actual membrane (Fig 5A, extracted from Fig 3O) we calculated D = 1.70, while for the simulated one we obtained D = 1.66 and 1.56 for G = 5 MPa and G = 7.5 MPa, respectively. The fractals observed in the actual experiments (5a) exhibit finer features compared to the ones obtained by the simulations (5b and 5c). This might be due to the constitutive model used and, in particular, the neighborhood (*N*_*x*_) radius *δ*. While the ratio *δ* = 3*h* we use in this work is typical for macroscopic problems [7], it may not be appropriate at the microscale. This will be a focus of future study. Our focus is to show the *transition* from the floral to the fractal morphologies. Finally, we note that fractals actually cannot truly exist in biomembranes, since the scaling behavior of natural objects is limited when approaching the molecular size region. Here, we assumed an approximately constant scaling behavior in the size range in which the biomembrane ruptures occur, even though it has been pointed out in the literature that it might be difficult to define the interval of scales over which the investigated structure displays consistent scaling behavior[39].

## Discussion

In this paper we have investigated numerically, by means of peridynamic simulations, ruptures in biomembranes. First, our numerical results indicate that the ruptures originate at the pinned regions; which in the experiments can be formed due to the bridging of Ca^2+^ in between the two stacked bilayers. In fact, the obtained rupture morphologies show a positional overlay of the pre-determined pinning points with the nucleation sites for the ruptures. The relationship between defects and the occurrence of fractures is common, and well established for solid materials[40]. Our findings confirm the earlier formulated presumption that for thin biomembrane sheets a similar dependency exists. Pinning sites perturb the continuity of the material structure, and respond in that sense very much like common solid materials.

An important result in our work is the transition of the pore morphology from floral to fractal with increasing shear modulus. Generally, fingering instabilities are associated with liquids, and in analogy, pore formation with fluid membranes, while fracture is associated with solids. In our experimental circumstances, lipid membranes, consisting of soy bean polar extract, are mainly in their liquid state while spreading and rupturing. This can be confirmed by the observation that after fracturing, the lipid membrane patches continue to spread[1], (see S3 Movie). However, as mentioned earlier, the membrane might still locally display partially increased rigidity caused by the presence of Ca^2+^ ions in between the distal and the proximal bilayers. Ca^2+^ is known to efficiently bind to lipid molecules, in particular to phosphatidyl choline (PC), a main constituent of soy bean polar extract [41]. In our experiments, the ambient buffer solution contains 4 mM Ca^2+^, which could leak into the MLV lamellae through defects during spreading, or through incisions formed during rupturing. Incubation of Ca^2+^ in the mM concentration range with lipid membranes has been shown to significantly decrease membrane fluidity[42–43]. Ca^2+^ has been also shown to strongly pin (interconnect) two stacked bilayers together [44]. The rigidity caused by the Ca^2+^-mediated pinning of the distal bilayer to the proximal is similar to the pinning found in biological cells, where tiny regions of the plasma membrane are anchored to the underlying cytoskeleton. Such attachment of the membrane has been shown to result in phase separation, i. e., nano- or micro- scale segmentation of membrane into coexisting disordered (liquid) and ordered (rigid) regions, the latter often referred to as 'gel phase'. The existence of many small rigid regions within a liquid membrane may lead to percolation, sub-diffusive behavior and shear response[45]. The dynamics of spreading membranes on solid supports is mainly dominated by friction rather than viscosity[23]. Friction in conjunction with local Ca^2+^-induced rigidity can also hinder membrane flow and promote shear response in the distal bilayer. Furthermore, handle-like defects bridging the two stacked bilayers involve inter-monolayer sliding[46] and friction, which would contribute to the shear response of the membrane. The analogy between the rupture propagation in membranes and the viscous fingering instabilities has previously been established[1]. In addition, purely elastic, non-viscous fingering instabilities in a Hele-Shaw model cell have recently been reported[47].

While we do not yet capture the dynamics of the fractal rupturing with these peridynamics simulations, we believe we can address satisfactorily the factors which determine the fundamental pore morphologies. The peridynamic approach provides a novel framework for continued interdisciplinary study in this area. In particular, determining a more accurate constitutive model for lipid membrane deformation and fracture is a relevant area that merits further research. We envision the technique to evolve to a state where rupture formation, timing and dynamics can be reliably predicted.

## Supporting Information

S1 Fig Rupture patterns for varying values of mass scaling. Peridynamic simulations (G = 5MPa) showing fractal ruptures for a mass scale of (a) 10^6^, (b) 10^4^, and (c) 10^2^.
(TIF)

S1 File Supplementary document containing Peridynamic mathematical detail and discussion of effect of mass scaling as well as S1 Fig. (PDF)

S1 Movie Microscopy time series of the rupturing in Fig 3A–3C. (MP4)

S2 Movie Microscopy time series of the rupturing in Fig 3G–3I. (MP4)

S3 Movie Microscopy time series of the rupturing in Fig 3M–3O. (MP4)
