# Supplementary figures and images for: Peridynamic Modeling of Ruptures in Biomembranes
Source: PLoS One. 2016 Nov 9;11(11):e0165947. doi: 10.1371/journal.pone.0165947 (PMC5102442; doi:10.1371/journal.pone.0165947)

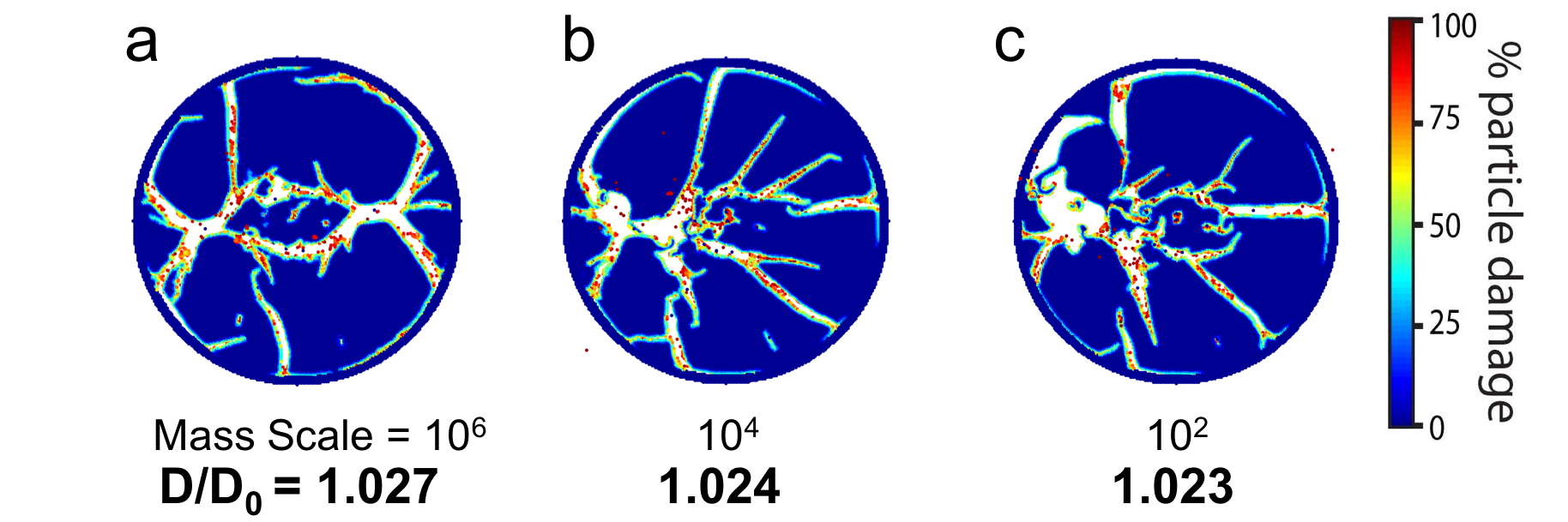

Supplement: S1 Fig — Peridynamic simulations (G = 5MPa) showing fractal ruptures for a mass scale of (a) 106, (b) 104, and (c) 102. (TIF) [file pone.0165947.s001.tif]
