# Supplementary material for: Peridynamic Modeling of Ruptures in Biomembranes
Source: PLoS One. 2016 Nov 9;11(11):e0165947. doi: 10.1371/journal.pone.0165947 (PMC5102442; doi:10.1371/journal.pone.0165947)
Supplement: S1 File — (PDF) [file pone.0165947.s002.pdf]

Peridynamic Modeling of Ruptures in Biomembranes

SUPPORTING INFORMATION

Peridynamic theory

A peridynamic body is composed of a continuum of particles (material points). A given particle, initially located at x is bonded to a certain number of its nearest neighbors, x', within a specified spherical neighborhood of finite radius(Fig. 1). The equation of motion for the body at is given by[1]

$$\int_{N_x} \left\{\underline{\mathrm{T}}\langle x' - x\rangle - \underline{\mathrm{T}}'\langle x - x'\rangle\right\} dV_{x'} + \rho b = \rho \ddot{u} \qquad \text{(Eq. SI 1)}$$

where ρ is the mass density,b is the body force, N_x is the neighborhood of a point x(Fig. 1d), and $\underline{\mathrm{T}}$ is called the force vector state(Fig. 1g). The notation $\underline{\mathrm{T}}\langle x' - x\rangle$, for example, denotes that the force vector state is a mapping of the initial distance vector $x' - x$ to a traditional force vector. The displacement of particle x is given by u.

Let $\xi = x' - x$. A deformation maps this vector into $\eta = y' - y$. The bond extension is given by

$$\underline{e}\langle\xi\rangle = \|\eta\| - \|\xi\|. \qquad \text{(Eq. SI 2)}$$

The deviatoric part of the extension is

$$\underline{e}^d\langle\xi\rangle = \underline{e}\langle\xi\rangle - \frac{\theta\|\xi\|}{3}, \qquad \text{(Eq. SI 3)}$$

where the dilation θ is

$$\theta = \frac{3}{m}\int_{N_x} \underline{\omega}\,\langle\xi\rangle \underline{e}\langle\xi\rangle\|\xi\| d\xi;\, m = \int_{N_x} \underline{\omega}\langle\xi\rangle\, \|\xi\|^2 d\xi, \qquad \text{(Eq. SI 4)}$$

and $\underline{\omega}\langle\xi\rangle$ is the influence function. In this work we use $\underline{\omega}\langle\xi\rangle = |\xi\|^{-1}$[2].
We assume that the lipid membranes are reasonably well modeled by the linear force vector state[3]

$$\underline{T}\langle\xi\rangle = \left(\frac{3\kappa\theta}{m} + \frac{15G}{m} e^d\langle\xi\rangle\right) \underline{\omega}\langle\xi\rangle \underline{\mathrm{M}}\langle\xi\rangle, \qquad \text{(Eq. SI 5)}$$

where $\underline{\mathrm{M}}\langle\xi\rangle$ is a unit vector co-linear with the distance vector ξ, κ is the bulk modulus and μ is the shear modulus. In the case of infinitesimal strains, this material corresponds to a classical isotropic elastic solid. Taking $G = 0$ (i.e., no shear resistance) corresponds to the case of a simple linear fluid.

Effect of Mass Scaling

Due to the quasi-static nature of the applied loading, we use mass scaling to increase the stable time-step size in our numerical integration. As described in the main article, we use the implicit trapezoidal rule with fixed point iteration to solve for the particle displacements at each step. The algorithm is adaptive such that time-steps are chosen automatically to achieve a convergence tolerance of 10^{-6} in a maximum of 8 iterations. Without mass scaling, stable time-steps are determined to be no larger than approximately $10^{-9}s$, and often as small as $10^{-11}s$. Given the time scale of the systems we're analyzing, this is a prohibitively small value. Using a mass scale of 10^{8} allows for tractable stable time-steps of approximately $10^{-4}s$ prior to rupture and $10^{-6}s$ during rupture.

To see the effect of this scaling on the rupture patterns, we performed simulations for a shear modulus of 5GPa, varying the mass scaling from 10^{2} to 10^{8}, using the pinning pattern shown in Fig. 3p in the main text. Unfortunately, eliminating the mass scaling completely results in a simulation (wall clock) time estimated to be on the order of weeks and, thus, this was not included in this study. Snapshots of the rupture pattern for three representative scalings are shown in S1 Fig1. In comparison with scaling used in the main article (see Fig. 3p-r), reducing the mass scale causes ruptures to form slightly earlier, i.e., at smaller values of radial expansion, and nucleate at different pinned particles. However, qualitatively, the overall rupture patterns remain quite similar and retain their fractal nature. We suspect that the short-range force could be adjusted in each case to reduce the differences, but this is outside the scope of the current investigation. Given our primary focus on the qualitative aspects of lipid membrane rupture and the transition between rupture states, we consider the mass scaling used in the simulations to be appropriate.

S1 Fig1 Rupture patterns for varying values of mass scaling. Peridynamic simulations (G = 5MPa) showing fractal ruptures for a mass scale of (a) 10^{6}, (b) 10^{4}, and (c) 10^{2}.

[1] S. A. Silling, M. Epton, O. Weckner, J. Xu, and E. Askari, Journal of Elasticity **88**, 151 (2007).
[2] M. L. Parks, R. B. Lehoucq, S. J. Plimpton, and S. A. Silling, Computer Physics Communications **179**, 777 (2008).
[3] S. A. Silling, Journal of the Mechanics and Physics of Solids **48**, 175 (2000).
